# Near-fatal cocaine intoxication in an infant with thrombotic microangiopathy associated with multiple organ failure

**DOI:** 10.1590/1984-0462/2024/42/2022159

**Published:** 2023-08-25

**Authors:** Alejandro Donoso Fuentes, Gianfranco Tomarelli Rubio, Camila Ampuero Acuña, Franco Díaz Rubio, Fernando Bracho Milic, Pamela Carrasco Troncoso

**Affiliations:** a Hospital Clínico Dra. Eloísa Díaz I., Pediatric Critical Care Unit, Santiago, Chile.

**Keywords:** ADAMTS13, Cocaine, Drug-induced thrombotic microangiopathy, Pediatrics, Plasmapheresis, Thrombotic microangiopathies, ADAMTS13, Cocaína, Microangiopatia trombótica induzida por drogas, Pediatria, Plasmaférese, Microangiopatias trombóticas

## Abstract

**Objective:**

To report a pediatric case of drug-induced thrombotic microangiopathy caused by cocaine

**Case description:**

We report a nine-month-old patient who developed thrombotic microangiopathies after extreme cocaine intoxication, multiple organ dysfunction syndrome with hemodynamic dysfunction, anuric renal failure, liver failure, encephalopathy, and myocardial injury, corresponding phenotypically to thrombocytopenia-associated multiple organ failure. The patient received continuous venous hemofiltration and therapeutic plasma exchange, recovering satisfactorily. She was discharged after 30 days of hospitalization under the guidance of the childcare service, and was healthy after one year of follow-up. Toxicological samples confirmed high levels of cocaine and derivatives in blood, urine and hair.

**Comments:**

To our knowledge, this is the first reported pediatric case. There are particularities of cocaine intoxication pathophysiology that can trigger thrombotic microangiopathies because of vasoconstriction, direct endothelial injury, platelet activation, and increasing von Willebrand factor and fibrinogen levels. All of which results in a prothrombotic state, inflammatory dysregulation, and microvascular thrombi. The increasing use of cocaine, especially among young adults, puts children at high risk of toxicity, either by passive unintentional exposure, or abuse due to the increased availability in homes.

## RESUMO

**Objetivo:**

Relatar um caso pediátrico de microangiopatia trombótica induzida por drogas causada por cocaína

**Descrição do caso:**

Relatamos uma paciente de nove meses de idade que desenvolveu microangiopatia trombótica após intoxicação extrema por cocaína, síndrome de disfunção de múltiplos órgãos com disfunção hemodinâmica, insuficiência renal anúrica, insuficiência hepática, encefalopatia e lesão miocárdica, correspondendo fenotipicamente à falência múltipla de órgãos associada à trombocitopenia. A paciente recebeu hemofiltração venosa contínua e plasmaférese terapêutica, recuperando-se satisfatoriamente. Recebeu alta após 30 dias de internação sob orientação do serviço de puericultura e estava saudável após um ano de seguimento. Amostras toxicológicas confirmaram altos níveis de cocaína e derivados no sangue, urina e cabelos.

**Comentários:**

Até onde sabemos, este é o primeiro caso pediátrico relatado. Existem particularidades da fisiopatologia da intoxicação por cocaína que podem desencadear a microangiopatia trombótica devido à vasoconstrição, lesão endotelial direta, ativação plaquetária e aumento do fator de von Willebrand e dos níveis de fibrinogênio. Tudo isso resulta em um estado pró-trombótico, desregulação inflamatória e trombos microvasculares. O uso crescente de cocaína, principalmente entre adultos jovens, coloca as crianças em alto risco de toxicidade, seja por exposição passiva não intencional ou abuso devido à maior disponibilidade nas residências.

## INTRODUCTION

Microangiopathic hemolytic anemia (MAHA) refers to hemolytic anemia related to red blood cell fragmentation associated with small vessel disease. Thrombotic microangiopathy (TMA) describes syndromes characterized by MAHA, thrombocytopenia, and thrombotic lesions in small blood vessels. The most common diagnoses associated with TMA are hemolytic uremic syndrome (HUS) in children and thrombotic thrombocytopenic purpura (TTP) in young adults. Still, it may develop due to many different disorders, including adverse reactions to drugs.^1^

Drug-induced TMA (DITMA) has also been reported in association with exposure to illegal substances.^2^ The most frequent stimulant illicit drug in Western countries is cocaine, but reports on DITMA and cocaine are scarce.^3–6^

Young adults are the primary users of cocaine, therefore, passive and unintentional exposure is a particular concern in infants and young children, given the negative short- and long-term outcomes.^6^

Regards acute cocaine toxicity, thrombocytopenia-associated multiple organ failure (TAMOF) or DITMA have not been previously described in children.

We report an infant with acute cocaine poisoning that developed severe cocaine-induced TMA and the unusual TAMOF phenotype, requiring respiratory and hemodynamic support, and therapeutic plasma exchange, with a favorable outcome.

## CASE REPORT

A previously healthy nine-month-old female, 10 kg, was brought to the pediatric emergency room due to generalized tonic-clonic movements. At admission, her temperature was 38.6°C, her pulses were weak, and the electrocardiogram (EKG) showed a monomorphic ventricular tachycardia of 204 beats/min. She received synchronized electrical cardioversion (1 J/kg), and an endotracheal tube was placed, recovering sinus rhythm. Afterwards, she was quickly transferred to the Pediatric Intensive Care Unit (PICU). Upon admission, the patient was tachycardic, 187 beats/min, her blood pressure was low (73/34 mmHg), and her core body temperature was 39.4°C. Nutrition status: overweight.

It was remarkable on physical exam, reactive mydriatic pupils, poor hygiene, and neglect signs. A sympathomimetic toxidrome was suspected due to the hyperadrenergic state and presumptive cocaine exposure. Immunochromatographic rapid test for drug screening in urine confirmed cocaine (cutoff point >300 ng/ml). On direct questioning of the mother, she established the availability of cocaine at her residence. Body packing was not suspected; a plain abdominal X-ray ruled out the presence of a foreign body in the stomach, rectum, or colon.

Once admitted to PICU, the patient received mechanical ventilation, inotropic and vasoactive support (epinephrine and norepinephrine), neuroprotection measures (including anticonvulsants), and empirical antibiotic therapy.

Within the first 24 hours of admission, the patient developed multiple organ dysfunction syndrome (MODS). She had hemodynamic dysfunction (lactate 18.3 mmol/L, troponin I 2338 pg/mL), anuric acute renal failure (creatinine increased from 0.42 mg/dL on admission to 1.14 mg/dL), liver failure, coagulopathy (D-dimer 20853 ng/mL, prothrombin time [PT] 34%) and encephalopathy. Initial laboratory tests are summarized in Table 1. An EKG demonstrated sinus rhythm without signs of myocardial ischemia, and the echocardiogram was normal at the time. Brain magnetic resonance imaging was normal. Renal ultrasound showed patent intraparenchymal renal arteries and resistance index not exceeding 0.7. Due to progression to anuric acute renal failure, it was decided to start renal replacement therapy with venous hemofiltration.

**Table 1:** Temporal evolution of the main laboratory tests.

Parameter		Reference range	Admission	24–48 h	4–5 days	15 days
Complete blood count						
	Hemoglobin (g/dL)	10.5–13.5	11.5	7.3	7.8	9.3
	Hematocrit (%)	33–39	36	21.3	22	28
	WBC count (x103/mm^3^)	6–17.5	20.2	8.3	5.0	7.8
	Platelet count (x103/mm^3^)	140–400	377	130	460	343
Coagulation markers						
	PT (%)/INR	70–130	34/2	26.5/2.5	84/1	86.9/1
	aPTT (sec)	26.4–37.5	-	57.1	-	-
	Haptoglobin (mg/dL)	41–165	-	-	28.9	-
	D-dimer (ng/mL)	0–550	20,853	19,963	15,704	1,234
	ADAMTS13 activity assay (%)	41–18	-	-	45	96
Chemical profiles						
	LDH (U/L)	195–349	344	1,633	644	298
	Total bilirubin/conjugated (mg/dL)	0.3–1.2	< 0.2/-	0.3/0.2	0.5/0.3	0.5/0.3
	Aspartate aminotransferase (U/L)	28–57	74	3,270	637	47
	Alanine aminotransferase (U/L)	15–35	54	2,025	1,223	84
	Alkaline phosphatase (U/L)	163–427	269	158	209	86
	Ferritin (ng/mL)	10–291	5,069	4,229	506	-
Renal						
	Blood urea nitrogen (mg/dL)	9–22	11	17	13	13
	Creatinine (mg/dL)	0.36–0.63	0.42	1.14	1.5	0.32
	Cardiovascular
	Troponin I (pg/mL)	0–38.6	2,338	474	32	-

g/dL: grams per deciliter; WBC: white blood cell; mm^3^: cubic millimeters; PT: prothrombin time; INR: international normalized ratio; aPTT: activated partial thromboplastin time; mg/dL: milligrams per deciliter; ng/mL: means nanograms per deciliter; ADAMTS13: A disintegrin-like and metalloprotease with thrombospondin type 1 motif n° 13; LDH: lactate dehydrogenase; U/L: units per liter; pg/mL: picograms per deciliter.

The next day laboratory findings evidenced exacerbation of anemia with signs of ongoing microangiopathic hemolytic anemia (hemoglobin decreased from 11.5 to 7.3 g/dL), lactate dehydrogenase (LDH) increased from 344 to 1633 U/L, and haptoglobin was 28.9 mg/dL (normal value [NV]: 41–165 mg/dL) with the persistence of high levels of D-dimer. Direct antiglobulin test (DAT) was negative, and no schistocytes in peripheral blood smear were seen. There was a decrease in platelet count (130 k/mm^3^) (Table 1) and an increase in encephalopathic compromise, not attributable to metabolic causes or residual pharmacological effect. Additionally, the PT decreased to 26.5%, with prolongation of the activated partial thromboplastin time (aPTT) to 57.1 seconds (NV: 26.4–37.5 sec). TMA with TAMOF phenotype, DITMA, or TTP were presumptive diagnoses. ADAMST13 was not available at our center but was urgently requested. PLASMIC score was 6. Due to progressive deterioration, plasma exchange therapy was decided as empirical therapy, even considering the patient's low-risk PLASMIC score. A 7-Fr, double-lumen catheter (Soft-Line^®^ Catheter, Medcomp^®^, Harleysville, PA, USA), was placed in the femoral vein for therapeutic plasma exchange (TPE). The apheresis procedure was performed with Prismaflex eXeed™ II equipment (Gambro Lundia, Lund, Sweden), using a TPE-1000™. One volemia was exchanged daily for four days, using fresh frozen plasma as replacement fluid. She had a complete response to the therapy according to Paglialonga criteria.^7^

She improved her mental status, normalizing LDH, increasing platelet count (>150 k/mm^3^ at 48 hours), and recovering diuresis, renal and hemodynamic function. The ADAMTS13 (disintegrin-like and metalloprotease with thrombospondin type 1 motif n° 13) enzyme study was not characteristic for idiopathic TTP, with an activity of 45% (NV: 41–180%), and the absence of an inhibitor. The patient's clinical condition progressively improved, receiving two days of renal replacement therapy and eight days of mechanical ventilation. The length of stay in the intensive care unit was 17 days.

The examination of three biological matrices found extremely high levels of cocaine, 0.57 mg/L in blood and 15.021 ng/mL in urine, 112.2 ng/mg of cocaine and 10.37 ng/mg of benzoylecgonine in hair analysis, compatible with chronic cocaine exposure. Legal actions were taken by child services and hospital administration for child abuse. However, it was never possible to determine the specific exposure route and the amount of cocaine received by the infant in the hours before her hospital admission. Genetic testing of thrombotic microangiopathies was not performed.

The patient fully recovered, being discharged after a month of hospitalization without morbidities acquired during the PICU stay and without functional sequelae or clinical symptoms at one year of follow-up

## DISCUSSION

Endothelial cell injury is one of the main features in the events that cause TMA. Therefore, it seems reasonable to attribute the direct toxic effect of cocaine on the endothelium as the primary driver of TMA in the presented case, given the patient's age. In particular, the clinical course of the patient and the development of DITMA are probably due to acute toxicity caused by a very high dose received on a previously existing vascular damage (chronic toxicity). Thus, the massive acute exposure to the drug triggered extensive and severe endothelial damage and secondary microvascular involvement. Consequently, the pathogenic mechanisms proposed for the development of DITMA depend on the characteristics of the toxic substance and the dose and duration of exposure.^1^

**Figure 1:**
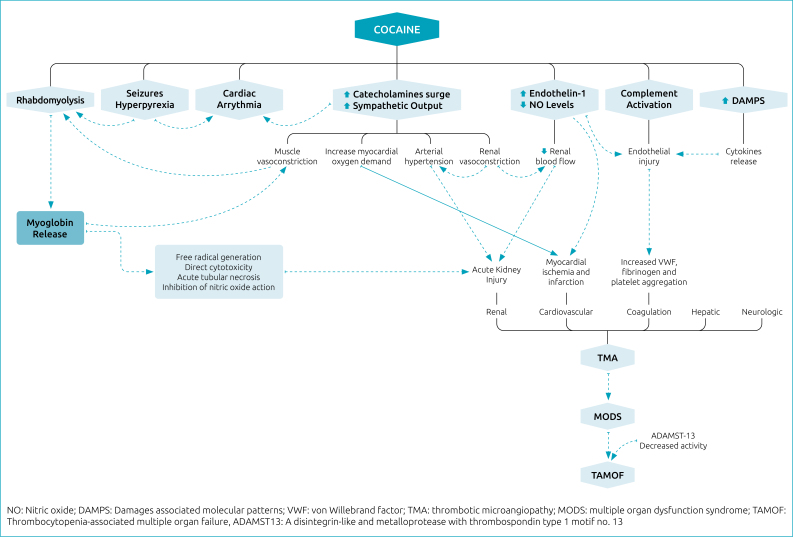
Mechanisms and pathophysiological pathways of acute and chronic cocaine effects leading to multi-organ involvement.

Experimental and clinical studies found a close relation between cocaine exposure and endothelial dysfunction, being the main responsible for the increased incidence of cardiovascular complications among users. Exposure to cocaine increases the production of endothelin-1 and decreases the expression of endothelial nitric oxide synthase (eNOS), all of which result in the development of vasoconstriction and endothelial activation.^8^ In addition, the endothelial stimulation promotes an increased activity of circulating factor VIII and unfractionated large von Willebrand factor (VWF) due to endothelial exocytosis, both with prothrombotic activity. Unfractionated large VWF multimers are cleaved by the ADAMTS13 enzyme into smaller and normal active VWF multimers; thus, in the presence of an unbalanced excess of VWF, ADAMTS13 is consumed, decreasing its circulating activity. This imbalance results in large unfractionated VWF anchoring to endothelial cells, tethering platelets, and initiating the development of microthrombi. This mechanism is postulated to be responsible for the formation of disseminated microthrombi, leading to circulatory dysfunction, ischemia, and organ failure.^9–11^ Many molecular pathways of cocaine-induced endothelial dysfunction amplify the dysregulated inflammatory response and the prothrombotic state, some of which are depicted in Figure 1.

In our opinion, unlike other cases, the magnitude of the various organic dysfunctions reported in this case was due to the prolonged exposure and unusually high levels of cocaine. For example, the drug concentration detected in the hair was more than double the median reported in adult daily users.^12^ In addition, although the clinical and laboratory findings were compatible with TMA, ADAMTS13 value was moderately decreased before TPE without the presence of an inhibitor, making a TTP less likely. Furthermore, the patient continued to improve after the suspension of plasma exchange supported a different alternative diagnosis, leaving the first possibility to consider a DITMA.

The observed ADAMTS13 values are consistent with the clinical phenotype of TAMOF. The TAMOF is currently recognized as a distinctive MODS phenotype, presenting a high mortality.^13,14^ It has an acquired of characteristic partial ADAMTS13 deficiency, a mild to moderate decrease in ADAMTS13 activity, with pathological findings of disseminated microvascular thrombosis,^13,14^ but hemolysis is usually not present.^15,16^

Recently, the role of hyperferritinemia in the induction of VWF factor secretion and the suppression of ADAMTS13 activity has been reported (increase in the VWF/ADAMTS13 ratio). This inflammatory biomarker showed a significant rise in the case reported herein.^17^

There are few papers of cocaine-induced MAHA/TMA to date.^2,18–23^ All of them are focused in adult patients, some associated with acute renal failure requiring dialysis, and the most recent ones treated with therapeutic plasma exchange and ADAMTS13 testing.^5,19,20^ Several publications have diverse positions on the treatment offered in each case, suggesting general supportive therapy. However, we opted to treat the patient with therapeutic plasma exchange, as recommended for TAMOF, given the underlying pathophysiological similarity, its life-threatening severity, availability of the therapy, and the treating team experience. According to the American Society for Apheresis (ASFA) guidelines, TAMOF is classified as category III for TPE indication, meaning that there is no definitive data of the beneficial role of apheresis.^24,25^

In the presented case, after four sessions of TPE, there was a complete response to the treatment according to expert guidelines.^7^

Regarding plasma exchange, beyond the mechanisms of removing large unfractionated VWF and replacing ADAMTS13 by donor plasma, apheresis may have reduced circulating cocaine metabolites that perpetuate and exacerbate DITMA. This may be especially relevant in infants and young children because the elimination of these compounds is slower compared to adults due to the lower activity of hepatic and plasma esterases.

Cocaine use in the general population, and especially young adults, has been steadily increasing worldwide over the last four decades, and more recently, in Latin America. Children, especially infants, are at high risk of unintentional exposure or direct exposure when associated with child abuse. We reported a case of severe acute over chronic exposure that developed DITMA and MODS and explored the rationale of early TPE. It is necessary to increase awareness about the severe and diverse forms of cocaine toxicity, especially considering chronic and subacute exposure. In addition, the different therapeutic options to be evaluated must be known, which must always be weighed individually in the patient.

## Data Availability

The database that originated the article is available with the corresponding author.
